# Age-Related Changes in Interaural-Level-Difference-Based Across-Frequency Binaural Interference

**DOI:** 10.3389/fnagi.2022.887401

**Published:** 2022-07-27

**Authors:** Matthew J. Goupell

**Affiliations:** ^1^Department of Hearing and Speech Sciences, University of Maryland, College Park, MD, United States; ^2^Neuroscience and Cognitive Science Program, University of Maryland, College Park, MD, United States

**Keywords:** binaural hearing, aging, hearing loss, interaural level differences (ILDs), across-frequency binaural interference

## Abstract

Low-frequency interaural time differences and high-frequency interaural level differences (ILDs) are used to localize sounds in the horizontal plane. Older listeners appear to be worse at horizontal-plane sound localization to compared younger listeners, but little is understood about age-related changes to across-frequency binaural processing. This study investigated if the frequency dependence of across-frequency ILD processing is altered for older compared to younger listeners, which was done by using an across-frequency binaural interference task (when the interaural difference sensitivity for a target sound is decreased by a spectrally remote interfering sound with zero interaural differences). It was hypothesized that as listeners experience advancing age and age-related high-frequency hearing loss (i.e., presbycusis), they will demonstrate worse binaural performance and experience more across-channel binaural interference (because of age-related temporal processing deficits), and will increasingly be affected by interferers at lower frequencies (because of age-related hearing loss) when compared to younger listeners. There were 11 older (>65 yrs) and 20 younger (<30 yrs) listeners with normal to near-normal audiometric thresholds up to 2 kHz. They were tested using a left-right ILD lateralization discrimination task. Single-tone ILD discrimination thresholds and across-frequency binaural interference were measured at 0.5, 1, 2, 4, and 8 kHz. ILD thresholds and interference were about twice as large for older compared to younger listeners. Interferers ≤1 kHz produced 2–3 times as much across-frequency binaural interference for older compared to younger listeners. Hearing thresholds were significant predictors of single-tone ILD thresholds; in addition, both target and interferer hearing thresholds were significant predictors of binaural interference. The results suggest a reweighting of binaural information that occurs with advancing age and age-related high-frequency hearing loss. This evidence of plasticity may help explain some of the age-related changes in spatial-hearing abilities.

## Introduction

The prevalence of background noise in social situations is high. Communicating in non-quiet and acoustically complex listening environments is facilitated greatly by spatial hearing, particularly in younger listeners. In comparison, older listeners demonstrate diminished spatial-hearing benefits in background noise and report greater difficulty communicating in these situations (Srinivasan et al., 2016; Gallun and Best, 2020; Gallun et al., 2021). A portion of age-related auditory processing deficits manifest from high-frequency hearing loss (i.e., presbycusis), another portion may manifest from central or neural age-related temporal processing deficits (Anderson et al., 2018, 2021), and another portion may manifest from cognitive processing (for reviews, see Working Group on Speech Understanding Aging, 1988; Gordon-Salant et al., 2010; Helfer et al., 2020).

Focusing on human sound localization in the horizontal plane, the “Duplex Theory of Sound Localization” (Strutt, 1907) states that interaural time differences (ITDs) at lower frequencies (less than ~1.5 kHz; Hartmann, 2021) and interaural level differences (ILDs) at higher frequencies are used for sound localization. The relative weighting of interaural information heavily favors low-frequency ITDs for sound localization (Wightman and Kistler, 1992; Macpherson and Middlebrooks, 2002). For pure tones presented in the free field, sound-localization and location-discrimination performance is a constant function of frequency, except that performance worsens between 1.5 and 3 kHz (Stevens and Newman, 1936; Mills, 1958). This may be because neither ITDs nor ILDs are conveyed well in this frequency region (Macaulay et al., 2010; Brughera et al., 2013). When presented over headphones, humans are sensitive to changes in ILDs at all frequencies and there is relatively equal ILD sensitivity across frequency (e.g., Brown and Tollin, 2021), despite the fact that ILDs are largest at higher frequencies (Feddersen et al., 1957; Hartmann, 2021). If there is an increase in ILD-based lateralization with increasing frequency, it is a small effect (Bernstein and Trahiotis, 2011; Goupell and Stakhovskaya, 2018b). The small or negligible ILD sensitivity frequency dependence is also consistent with physiological recordings (Jones et al., 2015). ILDs are first computed in the lateral superior olive of the superior olivary complex, and require temporally precise inputs on the order of milliseconds (Brown and Tollin, 2016; Franken et al., 2018; Ashida et al., 2021), similar to the temporal precision required of ITD processing in the medial superior olive (Goldberg and Brown, 1969; Yin and Chan, 1990).

The temporal precision to binaural neural encoding is affected by aging and hearing loss (Ross et al., 2007; Grose and Mamo, 2012; Ozmeral et al., 2016; Anderson et al., 2018; Eddins and Eddins, 2018; Eddins et al., 2018; Gallun et al., 2021). For example, sound localization in the vertical (Otte et al., 2013) and horizontal planes (Dobreva et al., 2011) appear to worsen with increasing age and hearing loss. ITD discrimination sensitivity (Strouse et al., 1998) and binaural temporal fine structure sensitivity (Eddins and Eddins, 2018; Füllgrabe and Moore, 2018; Füllgrabe et al., 2018) also decrease with increasing age. There appears to be stronger high-frequency rate limitations with increasing age for modulated high-frequency signals (Anderson et al., 2019). It could be that many of the binaural temporal processing deficits are partially related to age-related monaural temporal processing deficits (Grose and Mamo, 2012; Ihlefeld et al., 2015; Laback et al., 2017; Devries et al., 2022). Age-related decreases in ITD sensitivity and lateralization readily occur, whereas age-related decreases in ILD sensitivity and lateralization may be relatively smaller or negligible (Babkoff et al., 2002; Anderson et al., 2019).

While age-related performance degradation occurs across a wide array of hearing abilities, the brain might adapt as listeners lose access to different types of spatial information. Otte et al. (2013) hypothesized that listeners may shift their spectral filtering cue range for vertical plane localization from higher to lower frequencies with increasing age. The rationale included a functional role for listeners' pinna growing larger with age, which would physically shift the relevant cues to lower frequencies, where high-frequency age-related hearing loss would be less extreme. While their hypothesis was not supported, age-related changes to ITD and ILD processing for horizontal-plane localization can be similarly considered. On one hand, high-frequency age-related hearing loss would reduce access to the largest (Feddersen et al., 1957; Hartmann, 2021) and arguably most useful ILDs; on the other hand, age-related temporal processing deficits would reduce access to potent and heavily weighted low-frequency ITDs. Therefore, it may be that low-frequency ILD cues may become weighted more heavily because there is a greater reduction in ITD relative to ILD sensitivity (Eddins and Hall, 2010); however, this hypothesis concerning age-related changes to the frequency dependence of binaural processing has not yet been explored (Gallun, 2021).

Therefore, a study was performed to evaluate age-related changes to across-frequency ILD processing. This was done using an “across-frequency binaural interference” task (Mcfadden and Pasanen, 1976), where the listener is asked to discriminate or lateralize changing interaural differences in a target band and ignore the other remote and spectrally resolved interfering band that has fixed interaural information (i.e., there is conflicting binaural information across frequencies, such as a diotic or zero ILD interferer band). Interference occurs when the discrimination sensitivity or extent of lateralization decreases in the presence of the remote interferer, meaning the interaural information is combined across frequency channels at some point in the central auditory processing. These types of stimuli with a band with zero ILD and all bands with zero ITD are not experienced for natural sound sources outside the laboratory. One reason to use across-channel ILD processing is that it demonstrates a much stronger frequency dependence than occurs for single frequencies (Goupell and Stakhovskaya, 2018a; Rosen and Goupell, 2022); however, the across-channel ILD processing frequency dependence is not yet well-understood (Best et al., 2021). Another reason to use across-channel ILD processing is that the frequency dependence of ILDs are not as large and extreme as occurs for ITDs (Heller and Richards, 2010), which means that the frequency effects may be more inclined to reveal age-related changes. Finally, another reason to use across-channel ILD processing is that if there is an age-related shift of ILD weighting to lower frequencies, this shifts the relative importance to a region where ILDs produced by the head are naturally smaller, and hence possibly less beneficial for spatial-hearing tasks. It was hypothesized that binaural performance would be worse and binaural interference would increase for older compared to younger listeners because of central changes independent of frequency, possibly related to age-related temporal processing deficits because all binaural comparisons require temporal precision. In addition, it was hypothesized that relatively more interference would occur for the lower frequency targets (e.g., <4 kHz) because of the onset of age-related high-frequency hearing loss.

## Materials and Methods

### Listeners

Eleven older listeners (average age = 72 yr; age range = 67–80 yr; 8 females) were tested. All older listeners had relatively good hearing for their age with audiometrically normal to near-normal hearing at octave frequencies from 0.25 to 2 kHz, which was defined as ≤30 dB HL with interaural asymmetries ≤15 dB. Larger hearing thresholds were allowed at 4 and 8 kHz, with interaural asymmetries ≤20 dB at these frequencies. Because these listeners had relatively good hearing for their age, particularly at low frequencies, they were called older normal-hearing (ONH) listeners despite some having larger hearing losses at ≥4 kHz. An additional ONH listener started the experiment, but did not finish it and their data was omitted from the analysis.

**Figure 1:**
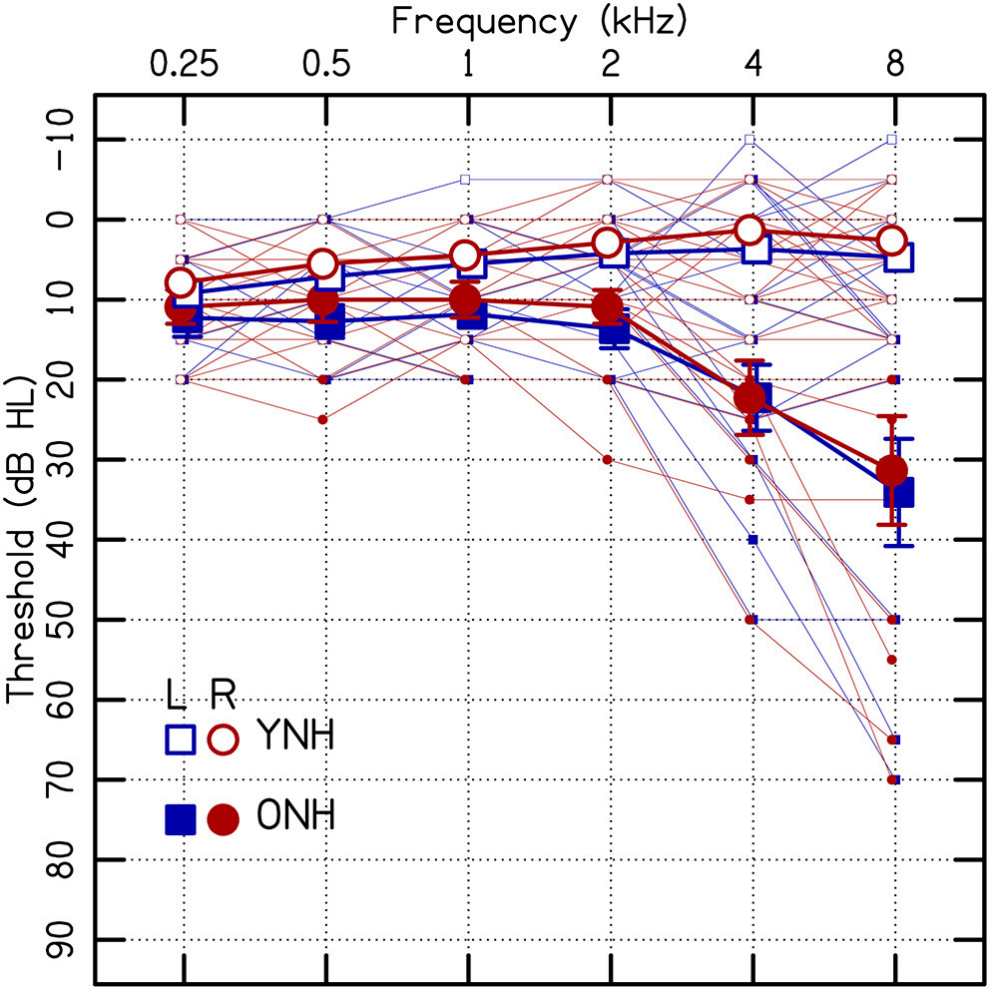
Individual (small points) and average (large points) audiometric thresholds in dB (re: hearing level, HL). Error bars represent ±1 standard error of the listener mean. Some points have error bars smaller than the size of the point. ONH, older normal hearing; YNH, younger normal hearing.

Twenty younger normal-hearing (YNH) listeners (average age = 22 yr; age range = 20–27 yr; 15 females) were tested as a control group; the data were previously reported in Rosen and Goupell (2022). They met stricter hearing threshold criteria: ≤20 dB HL with interaural asymmetries ≤15 dB at octave frequencies from 0.25 to 8 kHz. The average hearing thresholds for all the listeners are shown in Figure 1.

The ONH listeners also performed a cognitive screening test (Nasreddine et al., 2005). They all had a score of ≥22 (average = 26.9, S.D. = 2.5), indicating at most a mild cognitive impairment (Dupuis et al., 2015; Cecato et al., 2016). Therefore, poorer performance for the ONH listeners was not likely a result of cognitive impairment. A screening score from one ONH listener was not available because of experimenter error.

### Stimuli

The stimuli consisted of single target tones or target-interferer tone pairs presented at five frequencies of 0.5, 1, 2, 4, and 8 kHz. The tones were 300 ms in duration, had zero phase, and had a temporal raised-cosine ramp with a 10-ms rise-fall time applied. Tone pairs occurred simultaneously. Each tone was presented nominally at a level of 65 dB-A. Diotic level roving of ±10 dB (uniform distribution) was applied to the entire stimulus; in other words, the level roving did not introduce overall level differences across frequency. A non-zero ILD was applied to the target tones by increasing the level in dB in one ear by ILD/2 and decreasing the level in the other ear by ILD/2. The interfering tone was diotic, meaning it had zero ILD. Both targets and interferers had zero ITD. The target stimuli were audible and all listeners could perform the task for all conditions at these levels, even at 8 kHz, where some of the older listeners had high-frequency hearing loss. Stimuli had a sampling rate of 48.828 kHz. The stimuli were the same as in experiment 1 of Rosen and Goupell (2022).

### Equipment

The experiment was completed in a double-walled sound-attenuating chamber (Industrial Acoustics Inc.; Bronx, NY). Listeners were presented stimuli and the experiment was controlled using MATLAB (the Mathworks; Natick, MA) on a personal computer. The stimuli were delivered by a real-time processor, programmable attenuator, and headphone buffer (System 3 RP2.1, PA5, HB7; Tucker-Davis Technologies, Alachua, FL) to a pair of insert earphones (ER2; Etomotyic, Elk Grove Village, IL). Insert earphones minimized issues with ear canal collapse for the ONH listeners. The equipment was the same as in experiment 1 of Rosen and Goupell (2022).

### Procedure

Listeners controlled the experiment by interacting with a graphical user interface. They clicked a button to initiate a trial of an adaptive track. Each trial consisted of a two-interval left-right lateralization discrimination task. There was an interval that had a right-ear-favoring ILD and the other had a left-ear-favoring ILD, the direction of the ILD in the first interval randomly chosen with 50% *a priori* probability. The task was to report the perceived direction that the sound moved from the first to the second interval. After each trial, correct answer feedback was provided. The adaptive staircase procedure had 14 reversals. The ILD decreased if there were three sequentially correct responses and increased for an incorrect answer (79.4% correct on the psychometric function; Levitt, 1971). For 14 reversals, each track usually required 50–80 trials to reach completion. The starting target ILD for a track was 20 dB, a difference that was obvious to the listeners because the target tone move from one the side of the head to the other while the interferer tone remained near the center of the head; the correct answer feedback resolved any confusion about the identity of the target frequency. The ILD step size became smaller as the adaptive procedure continued. The ILD step size was ±4 dB for trials up to the second reversal, was ±2 dB for trials up to the fourth reversal, was ±1 dB for trials up to the sixth reversal, and was ±0.5 dB for the remaining trials. ILDs could not be higher than 20 dB or lower than 0 dB. If a listener had four incorrect responses at 20-dB ILD, the track was terminated immediately and the ILD threshold was recorded as 20 dB. This happened three times, once for one ONH listener and twice for another ONH listener. The arithmetic mean over the ILD values for the last 10 reversals of the adaptive track was used to calculate the left-right lateralization discrimination threshold per track.

Five target frequencies (f_tar_ = 0.5, 1, 2, 4, and 8 kHz) and five interferer frequencies (f_int_ = 0.5, 1, 2, 4, and 8 kHz) were tested. Control conditions tested the target frequencies in isolation. The across-frequency binaural interference conditions tested the target frequencies in the presence of a diotic (zero ITD and zero ILD) interferer, and every possible combination of the target and interferer frequencies was tested with the exception that the target and interference frequency could not be the same. At least three ILD thresholds were measured for each condition. This resulted in a total of 75 blocks in the experiment [5 f_tar_ × 5 f_int_ (including a no interferer control condition) × 3 blocks]. For one YNH listener who had more variability in their individual ILD thresholds, six thresholds were measured per condition because they had the time to do so. The order of conditions was randomized across the listeners and each condition was tested once before any condition was repeated, which helped minimize any order effects. Testing was ~6 h per listener, usually tested in 1- or 2-h sessions that occurred on 3–4 different days. Most listeners finished the study within 15 days, and one finished in 25 days. The procedure was the same as experiment 1 of Rosen and Goupell (2022).

### Analysis

Inferential statistics were performed using R version 4.0.3 (R Development Core Team, 2021). The *buildmer* (v2.3) (Voeten, 2020) and *lme4* (v1.1–26) (Bates et al., 2015) packages were used.

The first analysis was a linear mixed-effect model, which was performed for the single-frequency ILD discrimination thresholds and random intercepts were included for each listener. The dependent variable was the ILD discrimination threshold (in dB), where thresholds from the individual tracks were included to predict the average (i.e., listeners had at least three repeated measurements per condition). The independent categorical variables were age [2 levels: YNH (reference) and ONH] and frequency [f_tar_, 5 levels: 0.5, 1, 2, 4, and 8 kHz (reference)]. The rationale for using 8 kHz as the reference was that the greatest variation in hearing thresholds were at that frequency (Figure 1). An independent continuous variable, the interaural average of the hearing threshold (θ_tar_), was included as a frequency-specific predictor.

The second analysis started as a linear mixed-effect model, which was performed on the difference of the ILD thresholds in dB between conditions with no interferer and those with an interferer, called the binaural interference index (Bibee and Stecker, 2016). Random intercepts were included for each listener. The model building process with *buildmer*, which only includes terms that significantly improve the model, found that the random listener intercepts did not meet this requirement. Therefore, this analysis was reduced to a multiple regression. This analysis also used thresholds for individual tracks to predict performance. The independent categorical variables were age [2 levels: YNH (reference) and ONH], target frequency [f_tar_, 5 levels: 0.5, 1, 2, 4, and 8 kHz (reference)], and interferer frequency [f_int_, 5 levels: 0.5, 1, 2, 4, and 8 kHz (reference)]. Independent continuous variables included the interaural average hearing thresholds for the target (θ_tar_) and interferer (θ_int_) frequencies.

## Results

### Single-Tone ILD Discrimination Thresholds

**Figure 2:**
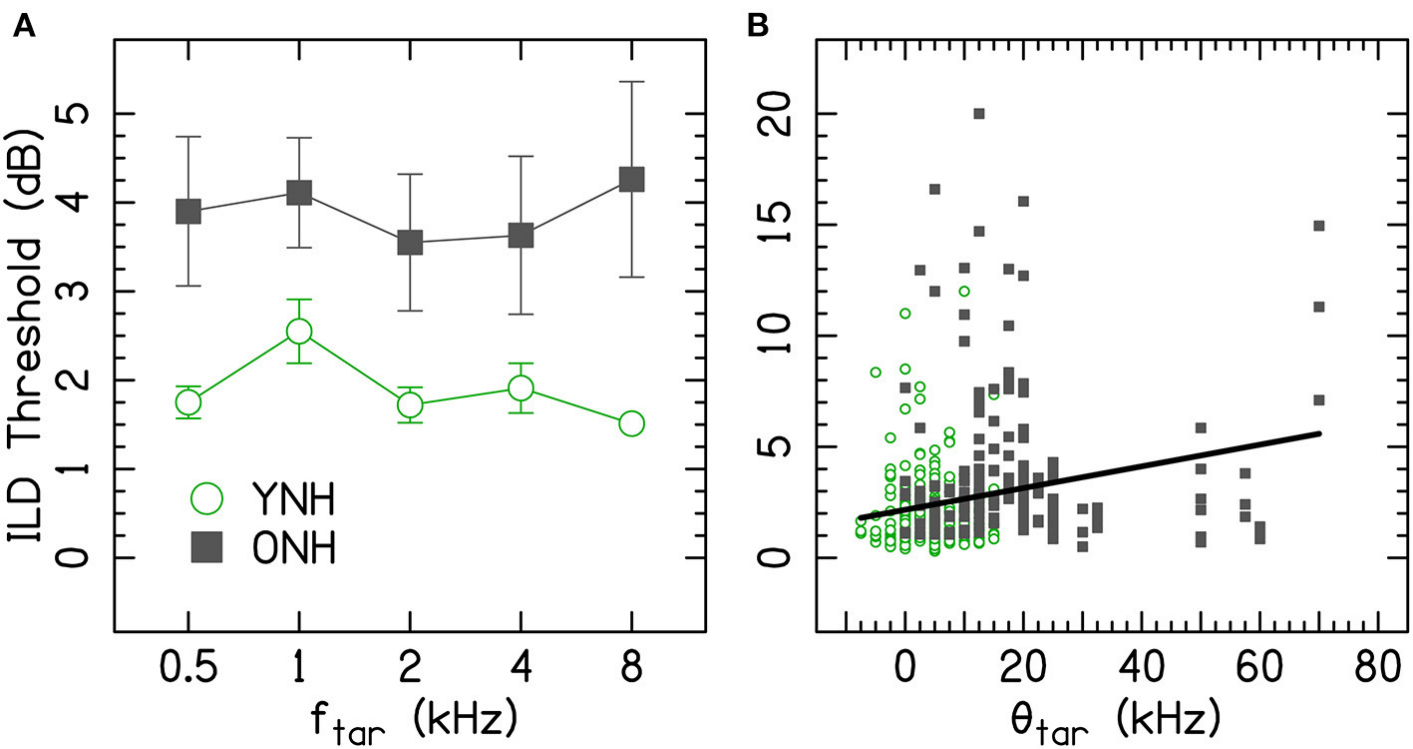
**(A)** Average ILD thresholds for single tones. Error bars represent ±1 standard error of the listener mean. **(B)** Individual ILD thresholds for single tones as a function of hearing threshold (θ_tar_). f_tar_, target frequency; ONH, older normal hearing; YNH, younger normal hearing; θ_tar_, average interaural hearing threshold at the target frequency. The YNH data have been previously reported. Reproduced from Beth Rosen and Matthew J. Goupell, “The effect of target and interferer frequency on across-frequency binaural interference of interaural-level-difference sensitivity,” J. Acoust. Soc. Am. 151, 924–938 (2022), https://aip.scitation.org/doi/10.1121/10.0009398, with the permission of Acoustical Society of America.

Figure 2 shows the average ILD thresholds for the conditions for the single tones (i.e., there was no diotic interferer at a remote frequency). The results of the linear mixed-effect model are reported in Table 1. The ONH listeners had significantly higher thresholds than YNH listeners (*p* = 0.020; Figure 2A), but there were no significant interactions with age. ILD thresholds increased with increasing θ_tar_ (*p* = 0.004; Figure 2B). The correlation between age and θ_tar_ was r = −0.23, suggesting that these were fairly independent effects for the listeners tested in this study. There was also a significant main effect of frequency, where worse ILD thresholds occurred at 1 kHz compared to 8 kHz (*p* = 0.001).

**Table 1:** Results of the linear mixed-effect model for the ILD thresholds for single tones.

**Fixed effects**	**Estimate**	**SE**	**df**	**t**	***p***	
**Intercept**	**1.431**	**0.418**	**47.0**	**3.43**	**0.001**	^**^
**Age(ONH)**	**1.555**	**0.636**	**32.1**	**2.44**	**0.020**	^*^
**θ_tar_**	**0.032**	**0.011**	**467.7**	**2.91**	**0.004**	^**^
f_tar_(0.5 kHz)	0.290	0.271	445.5	1.07	0.286	
**f**_**tar**_ **(1 kHz)**	**0.889**	**0.275**	**446.3**	**3.23**	**0.001**	^**^
f_tar_(2 kHz)	0.190	0.277	446.7	0.69	0.492	
f_tar_(4 kHz)	0.220	0.269	445.1	0.82	0.414	
Random effects	Variance	SD				
Listener (intercept)	2.50	1.58				
Residual	3.35	1.83				

Rows in bold highlight significant effects. Significance levels: ^*^p < 0.05, ^**^p < 0.01, ^***^p < 0.001. df, degrees of freedom; f_tar_, target frequency; ONH, older normal hearing; SD, standard deviation; SE, standard error; θ_tar_, average interaural hearing threshold at the target frequency.

### Across-Frequency Binaural Interference

**Figure 3:**
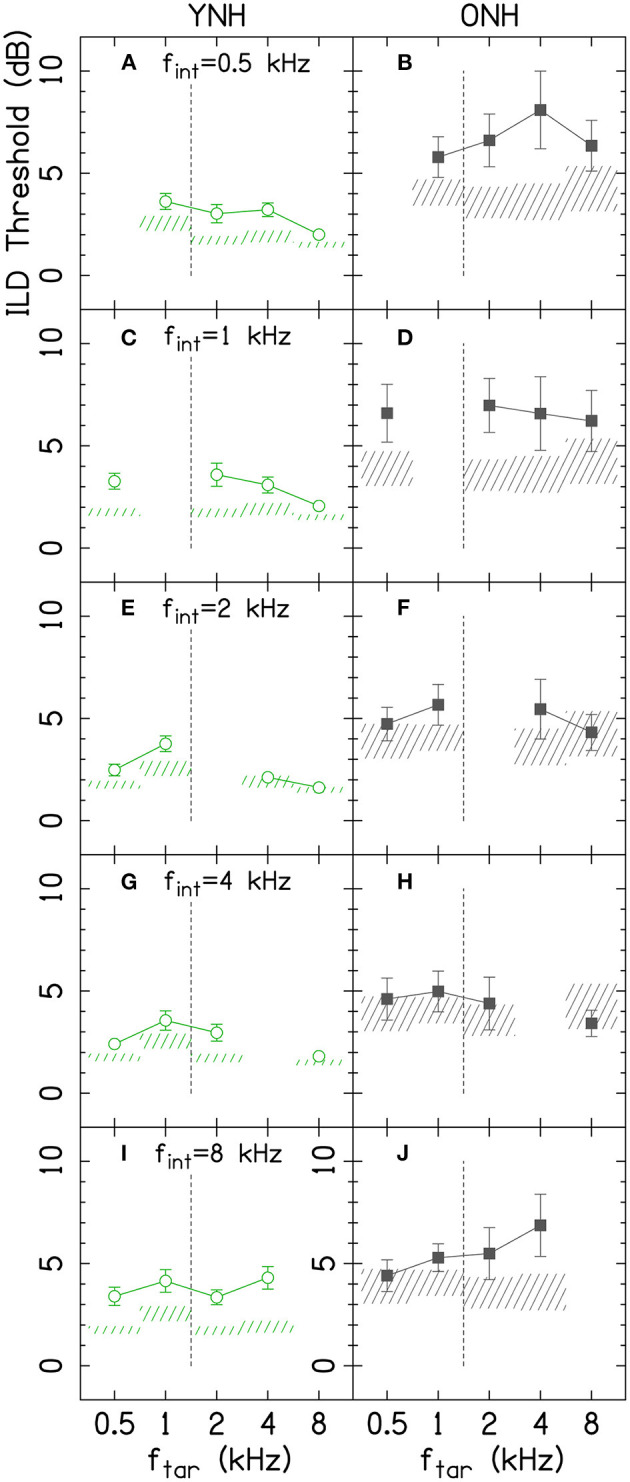
Average ILD discrimination thresholds for target-interferer pairs are shown by data points for YNH (left column) and ONH (right column) listeners. Different rows represent different f[^*int*^]. Error bars on each point represent ±1 standard error of the listener mean. The shaded areas represent the average ±1 standard error for the single-tone conditions, which were copied from Figure 2. The vertical dashed lines show the border where low-frequency ITDs are particularly potent. f_int_, interferer frequency; f_tar_, target frequency; ONH, older normal hearing; YNH, younger normal hearing. The YNH data have been previously reported. Reproduced from Beth Rosen and Matthew J. Goupell, “The effect of target and interferer frequency on across-frequency binaural interference of interaural-level-difference sensitivity,” J. Acoust. Soc. Am. 151, 924–938 (2022), https://aip.scitation.org/doi/10.1121/10.0009398, with the permission of Acoustical Society of America.

Figure 3 shows ILDs thresholds for the target-interferer pairs as a function of target and interferer frequency. The thresholds for the ONH listeners were generally higher than the thresholds for the YNH listener.

**Figure 4:**
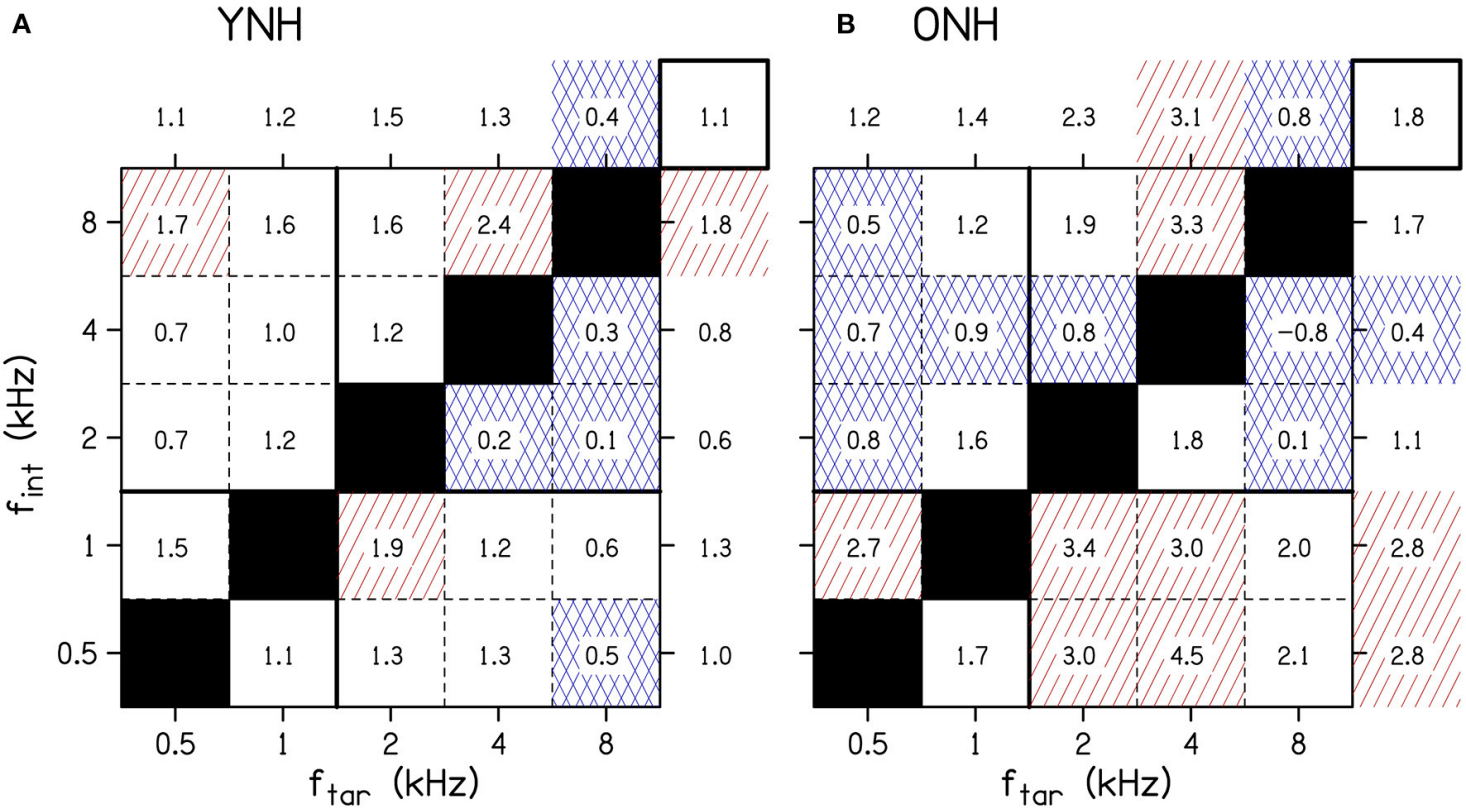
The average amount of binaural interference for YNH **(A)** and ONH **(B)** listeners. The numbers in the boxes represent the binaural interference index for target and interferer frequency combinations in dB. The numbers outside of the main grid are the average of each column and row. The overall average interference across all conditions is shown by the number in the top right box of each panel. Cells highlighted by red diagonal lines denote a relatively large amount of binaural interference, defined as at least 50% greater than the overall average. Cells highlighted by blue crossed lines denote a relatively small amount of binaural interference, defined as at least 50% less than average. The solid horizontal and vertical lines between 1 and 2 kHz show the border where low-frequency ITDs are particularly potent. f_int_, interferer frequency; f_tar_, target frequency; ONH, older normal hearing; YNH, younger normal hearing. The YNH data have been previously reported. Reproduced from Beth Rosen and Matthew J. Goupell, “The effect of target and interferer frequency on across-frequency binaural interference of interaural-level-difference sensitivity,” J. Acoust. Soc. Am. 151, 924–938 (2022), https://aip.scitation.org/doi/10.1121/10.0009398, with the permission of Acoustical Society of America.

To better understand the age-related and frequency-specific changes to the across-frequency binaural processing, Figure 4 shows the across-frequency binaural interference index (the difference between the target-interferer pair threshold and the target only threshold in dB from Figure 3) organized as a grid of target and interferer frequencies. The effects of frequency have some similar trends for the two age groups (e.g., f_tar_ = 1 kHz or f_int_ = 2 kHz), but there were also notable differences (e.g., f_tar_ = 0.5 kHz, f_int_ = 0.5 kHz, or f_int_ = 1 kHz).

The results of the multiple linear regression are shown in Table 2. The most important result from this analysis was that, depending on the frequency combination, the binaural interference index was larger for ONH listeners. Table 2 demonstrates numerous higher-order interactions with age, target frequency, and interferer frequency. These significant interactions support the hypothesis that across-frequency ILD processing changes with age, and that it changes in a frequency-specific manner.

**Table 2:** Main effects and significant interactions of the multiple regression for the binaural interference index (interactions that were not significant were omitted for clarity).

**Fixed main effects**	**Estimate**	**SE**	**t**	***p***	
**Intercept**	**3.190**	**0.834**	**3.825**	**0.0001**	^***^
Age(ONH)	−5.691	3.019	−1.885	0.060	
θ_tar_	−0.055	0.120	−0.461	0.645	
θ_int_	−0.154	0.134	−1.148	0.251	
f_tar_(0.5 kHz)	0.309	1.115	0.277	0.782	
f_tar_(1 kHz)	−0.929	1.065	−0.873	0.383	
f_tar_(2 kHz)	−0.686	0.977	−0.703	0.482	
f_tar_(4 kHz)	0.102	0.706	0.144	0.886	
**f_int_(0.5 kHz)**	**−2.783**	**1.115**	**−2.495**	**0.013**	^*^
**f_int_(1 kHz)**	**−2.723**	**1.065**	**−2.558**	**0.011**	^*^
**f_int_(2 kHz)**	**−2.728**	**0.639**	**−4.268**	**<0.0001**	^***^
**f_int_(4 kHz)**	**−2.708**	**0.935**	**−2.897**	**0.004**	^**^
Interactions					
**f_tar_(2 kHz) ×f_int_(1 kHz)**	**3.541**	**1.368**	**2.589**	**0.010**	^**^
**Age(ONH) ×θ_tar_**	**0.495**	**0.155**	**3.200**	**0.001**	^**^
**Age(ONH) ×θ_int_**	**0.573**	**0.246**	**2.328**	**0.020**	^*^
**Age(ONH) ×f_tar_(2 kHz)**	**7.871**	**3.499**	**2.250**	**0.025**	^*^
**Age(ONH) ×f_tar_(4 kHz)**	**5.633**	**2.536**	**2.221**	**0.026**	^*^
**Age(ONH) ×θ_tar_ ×θ_int_**	**−0.014**	**0.005**	**−2.783**	**0.005**	^**^
Age(ONH) ×θ_tar_ ×f_tar_(2 kHz)	**−0.480**	**0.214**	**−2.236**	**0.025**	^*^
**Age(ONH) ×θ_tar_ ×f_int_(0.5 kHz)**	**−0.399**	**0.165**	**−2.414**	**0.016**	^*^
**Age(ONH) ×θ_tar_ ×f_int_(1 kHz)**	**−0.504**	**0.166**	**−3.044**	**0.002**	^**^
**Age(ONH) ×θ_tar_ ×f_int_(2 kHz)**	**−0.450**	**0.136**	**−3.323**	**0.001**	^***^
**Age(ONH) ×θ_tar_ ×f_int_(4 kHz)**	**−0.420**	**0.174**	**−2.414**	**0.016**	^*^
**Age(ONH) ×θ_int_ ×f_tar_(2 kHz)**	**−0.575**	**0.254**	**−2.263**	**0.024**	^*^
**Age(ONH) ×θ_int_ ×f_tar_(4 kHz)**	**−0.662**	**0.230**	**−2.875**	**0.004**	^**^
**Age(ONH) ×θ_int_ ×f_int_(2 kHz)**	**−0.386**	**0.193**	**−2.003**	**0.045**	^*^
**Age(ONH)** **×f**_**tar**_**(1 kHz)** **×f**_**int**_**(2 kHz)**	**−8.688**	**3.446**	**−2.522**	**0.012**	^*^
**Age(ONH)** **×f**_**tar**_**(2 kHz)** **×f**_**int**_**(0.5 kHz)**	**−9.750**	**4.206**	**−2.318**	**0.021**	^*^
**Age(ONH)** **×f**_**tar**_**(4 kHz)** **×f**_**int**_**(0.5 kHz)**	**−11.903**	**3.802**	**−3.131**	**0.002**	^**^
**Age(ONH) ×θ_tar_ ×f_tar_(1 kHz) ×f_int_(0.5 kHz)**	**0.866**	**0.291**	**2.973**	**0.003**	^**^
**Age(ONH) ×θ_tar_ ×f_tar_(1 kHz) ×f_int_(2 kHz)**	**0.914**	**0.287**	**3.191**	**0.001**	^**^
**Age(ONH) ×θ_tar_ ×f_tar_(1 kHz) ×f_int_(4 kHz)**	**0.926**	**0.308**	**3.003**	**0.003**	^**^
**Age(ONH) ×θ_tar_ ×f_tar_(2 kHz) ×f_int_(0.5 kHz)**	**1.040**	**0.285**	**3.646**	**0.0003**	^***^
**Age(ONH) ×θ_tar_ ×f_tar_(2 kHz) ×f_int_(4 kHz)**	**0.729**	**0.288**	**2.530**	**0.011**	^*^
**Age(ONH) ×θ_tar_ ×f_tar_(4 kHz) ×f_int_(0.5 kHz)**	**0.375**	**0.157**	**2.387**	**0.017**	^*^
**Age(ONH) ×θ_int_ ×f_tar_(0.5 kHz) ×f_int_(1 kHz)**	**0.776**	**0.349**	**2.226**	**0.026**	^*^
**Age(ONH) ×θ_int_ ×f_tar_(2 kHz)×f_int_(1 kHz)**	**0.844**	**0.358**	**2.356**	**0.019**	^*^
**Age(ONH) ×θ_int_ ×f_tar_(4 kHz) ×f_int_(0.5 kHz)**	**0.999**	**0.329**	**3.034**	**0.002**	^**^
**θ_tar_ ×θ_int_ ×f_tar_(0.5 kHz)**	**0.022**	**0.008**	**2.645**	**0.008**	^**^
**θ_tar_ ×θ_int_ ×f_tar_(1 kHz)**	**0.021**	**0.009**	**2.480**	**0.013**	^*^
**θ_tar_ ×θ_int_ ×f_tar_(2 kHz)**	**0.031**	**0.009**	**3.553**	**0.0004**	^***^
**θ_tar_ ×θ_int_ ×f_tar_(4 kHz)**	**0.027**	**0.007**	**3.661**	**0.0003**	^***^
**θ_tar_ ×θ_int_ ×f_int_(1 kHz)**	**0.030**	**0.009**	**3.491**	**0.0005**	^***^
**θ_tar_ ×θ_int_ ×f_int_(2 kHz)**	**0.024**	**0.006**	**3.876**	**0.0001**	^***^
**θ_tar_ ×θ_int_ ×f_int_(4 kHz)**	**0.021**	**0.008**	**2.607**	**0.009**	^**^
**θ_tar_ ×θ_int_ ×f_tar_(0.5 kHz) ×f_int_(4 kHz)**	**−0.030**	**0.012**	**−2.572**	**0.010**	^*^
**θ_tar_ ×θ_int_ ×f_tar_(1 kHz) ×f_int_(0.5 kHz)**	**−0.044**	**0.019**	**−2.334**	**0.020**	^*^
**θ_tar_ ×θ_int_ ×f_tar_(1 kHz) ×f_int_(2 kHz)**	**−0.041**	**0.014**	**−2.881**	**0.004**	^**^
**θ_tar_ ×θ_int_ ×f_tar_(1 kHz) ×f_int_(4 kHz)**	**−0.033**	**0.010**	**−3.190**	**0.001**	^**^
**θ_tar_ ×θ_int_ ×f_tar_(2 kHz) ×f_int_(0.5 kHz)**	**−0.044**	**0.014**	**−3.086**	**0.002**	^**^
**θ_tar_ ×θ_int_ ×f_tar_(2 kHz) ×f_int_(4 kHz)**	**−0.040**	**0.011**	**−3.675**	**0.0002**	^***^
**θ_tar_ ×θ_int_ ×f_tar_(4 kHz) ×f_int_(0.5 kHz)**	**−0.042**	**0.011**	**−3.760**	**0.0002**	^***^

Rows in bold highlight significant effects. Significance levels: ^*^p < 0.05, ^**^p < 0.01, ^***^p < 0.001. f_int_, interferer frequency; f_tar_, target frequency; ONH, older normal hearing; SD, standard deviation; SE, standard error; θ_int_, average interaural hearing threshold at the interferer frequency; θ_tar_, average interaural hearing threshold at the target frequency.

There were the numerous significant four-way interactions that were difficult to fully interpret together in Table 2. Therefore, some of the four-way interactions and some of the more obvious lower-order interactions from the Figure 4 that include the factor age will be highlighted. When examining the columns of Figure 4, there were relatively larger binaural interference indices for ONH listeners at f_tar_ = 2 kHz [Age(ONH)×θ_int_×f_tar_(2 kHz), *p* = 0.024] and f_tar_ = 4 kHz [Age(ONH)×θ_int_×f_tar_(4 kHz), *p* = 0.004]. When examining the rows of Figure 4, there were larger binaural interference indices for ONH listeners at f_int_ = 0.5 [Age(ONH)×θ_tar_×f_int_(0.5 kHz), *p* = 0.016], 1 kHz [Age(ONH)×θ_tar_×f_int_(1 kHz), *p* = 0.002], and 2 kHz [Age(ONH)×θ_tar_×f_int_(2 kHz), *p* = 0.001]. There were smaller binaural interference indices for ONH listeners at f_int_ = 4 kHz [Age(ONH)×θ_tar_×f_int_(4 kHz), *p* = 0.016]. In addition, there were significant four-way interactions that depended on the target and interferer thresholds θ_tar_ and θ_int_, suggesting both play a role in the amount of across-frequency interference that occurred.

## Discussion

Aging and hearing loss affect spatial-hearing performance, but little is understood about age-related changes to across-frequency binaural processing and its frequency dependence. Binaural processing requires temporal precision, which degrades with increasing age. Using an across-frequency binaural interference task, where remote diotic (i.e., zero ILD and ITD) interferers often cause elevated target thresholds, ILD discrimination thresholds were measured as a function of target and interferer frequency in younger and older adult listeners, all having normal hearing thresholds up to 2 kHz. Across-frequency ILD processing shows relatively large effects of frequency compared to single tones (Goupell and Stakhovskaya, 2018a), but not the extreme asymmetry seen for ITD processing (Heller and Richards, 2010). The older listeners had relatively good hearing for their age, many with normal to near-normal thresholds, and increasing variability at 4 and 8 kHz where some had larger losses; nonetheless, they still had worse hearing threshold than their younger counterparts (Figure 1). It was hypothesized that aging would reduce binaural sensitivity and increase across-frequency binaural interference at all frequencies because of central changes, such as non-frequency-specific age-related temporal processing deficits. In addition, it was hypothesized that there would be relatively more interference caused by the lower frequency (e.g., <4 kHz) interferers because of the onset of age-related high-frequency hearing loss. These hypotheses were supported to some extent (Figures 3, 4; Table 2).

### Single-Tone ILD Discrimination Thresholds

The ILD discrimination thresholds for the single tones (i.e., the control conditions) were 3.9 dB on average for the ONH listeners, which was significantly higher than the 1.9 dB for the YNH listeners (Figure 2, Table 1). The significant increase in ILD discrimination thresholds for the ONH compared to the YNH listeners of the current study is larger than some other reports. For example, Herman et al. (1977) tested eight younger and eight older listeners with audiometrically normal hearing up to 2 kHz, and presented the listeners broadband clicks. They found no significant difference in ILD discrimination thresholds, the average threshold was 1.4 dB for the younger listeners and 1.5 dB for the older listeners. Babkoff et al. (2002) tested 78 listeners that had a range of ages from 22 to 88 yrs, had audiometrically normal hearing up to 2 kHz, and presented listeners broadband click trains. They also found no significant effects of age on intracranial lateralization and discrimination thresholds for ILDs.

Several studies have compared ILD discrimination thresholds for normal-hearing vs. hearing-impaired listeners, with some of the hearing-impaired listeners being older in age. These studies used narrowband noises (1/3rd-octave bandwidth), and include Gabriel et al. (1992; 2 of 4 hearing-impaired listeners >60 yrs), Koehnke et al. (1995; 2 of 11 hearing-impaired listeners >60 yrs), Smith-Olinde et al. (2004; some of 6 hearing-impaired listeners >60 yrs), and Spencer et al. (2016; 1 of 10 hearing-impaired listeners >60 yrs). In these studies, there was a small increase or no significant effect of hearing impairment on ILD thresholds. Therefore, the effect of age in the current study appears to be relatively larger than other similar studies.

One difference that might reconcile the different aging effects across studies is the stimuli that were used. The current study found a factor of two increase in ILD discrimination thresholds between age groups and used pure tones (a single frequency); the other studies found smaller or no increases and used wider bandwidth noise or click stimuli. Other studies have noted improved ILD and ITD performance with increasing stimulus bandwidth (Hartmann and Constan, 2002; Best and Swaminathan, 2019). In addition, ILD discrimination thresholds are worse for steady-state stimuli (like the tones used in the present study) compared to modulated stimuli (like noises) (Laback et al., 2017; Rosen and Goupell, 2022). Discrepancies in aging effects across studies such as for sound-localization performance (Dobreva et al., 2011; Otte et al., 2013) may also be related to differences in stimuli.

It is also possible to reconcile this relatively large perceptual result using tones in the current study with physiological findings. The relatively large aging effects could be a result of stronger neural adaptation for steady-state compared to modulated stimuli in older compared to younger listeners (Devries et al., 2022). Since physiological evidence for such age-related changes to the lateral superior olivary complex appear small (Casey, 1990; Finlayson and Caspary, 1993; Finlayson, 1995), such proposed changes may occur at higher centers (Laumen et al., 2016; Ashida et al., 2021).

Besides the age-related increase in ILD thresholds, there was a significant single-channel ILD frequency dependence, where 1 kHz had higher ILD thresholds than 8 kHz (Figure 2, Table 1). Other studies have also reported higher thresholds at 1 kHz in YNH listeners (Mills, 1960; Grantham, 1984; Yost and Dye, 1988; Goupell and Stakhovskaya, 2018a; Brown and Tollin, 2021; Rosen and Goupell, 2022); the reason for this increase may be a result of the interaction of a zero ITD cue on the ILD information (Brown and Tollin, 2021). There were no significant interactions between age and frequency to suggest the relative insensitivity at 1 kHz changes with age, although the frequency resolution of the current study may not have been appropriate to observe such a change. Any ILD frequency dependence has yet to be reconciled with neurophysiological recordings, which reveal little frequency dependence in lateral superior olivary neurons (Jones et al., 2015).

It is unclear why there is such a small ILD frequency dependence; some additional information from the present study contributes to better understanding this phenomenon. The average interaural hearing threshold was included as a frequency-specific predictor in the statistical model. The results in Table 1 revealed that ILD thresholds increased with increasing θ_tar_. The effect of average hearing threshold is perhaps unsurprising, given the numerous studies suggesting the need to control for differences in stimulation level in studies investigating aging and hearing loss (e.g., Durlach et al., 1981; Häusler et al., 1983; Smith-Olinde et al., 2004; Gallun et al., 2021). What is slightly more surprising is that the younger listeners had typical hearing thresholds and the older listeners had relatively good hearing for their age, with elevated hearing thresholds at only 4 and 8 kHz. Some reports have shown that audiometric thresholds are not a significant predictor of binaural performance (e.g., Gabriel et al., 1992; Koehnke et al., 1995), but they did not utilize statistical approaches that were well-suited to include frequency-specific hearing thresholds as a predictor. Many previous studies had too few listeners to test for correlations between hearing thresholds and binaural performance, although it is sometimes possible to observe elevated hearing thresholds to correlate with worse binaural performance. For example, Spencer et al. (2016) found a significant correlation between hearing thresholds and ILD thresholds at 4 kHz for normal-hearing listeners, but not at 0.5 kHz for normal-hearing listeners, not for hearing-impaired listeners, and not for other binaural processing measures like ITD and interaural correlation change discrimination sensitivity. On the other hand, even small increases in hearing thresholds within an audiometrically normal hearing range appear to diminish binaural sensitivity (Bernstein and Trahiotis, 2016, 2018) and alter across-frequency binaural interference patterns (Bernstein and Trahiotis, 2021).

In summary, based on the current study, it appears that the individual variation in single-channel ILD thresholds are attributed to both age and hearing sensitivity. Future studies could investigate this more thoroughly by intentionally recruiting listeners with a wide range of average hearing sensitivity and hearing asymmetry, and using statistical approaches like mixed-effect models to appropriately handle frequency-specific predictors of performance.

### Across-Frequency Binaural Interference

ILD thresholds increased in the presence of the interferer (Figure 3, compare points and shaded areas) and that amount changes depending on the target and interferer frequency. The amount of interference is most easily seen in Figure 4. The binaural interference indices were about twice as large for the older listeners (1.8 dB) compared to the younger listeners (1.1 dB; top-right corner box of each panel in Figure 4), which was not a significant increase [main effect of Age, *p* = 0.060; Table 2]. There were numerous higher-order interactions with age highlighting that the increases were specific to certain combinations of target and interferer frequencies.

To summarize the results for the younger listeners (Figure 4A), the least interference was experienced by the 8-kHz target tones and the most interference was produced by the 8-kHz interferer tones (see Rosen and Goupell, 2022 for a detailed summary). The amount of interference was not particularly well-predicted by the single-tone ILD thresholds. Much like the frequency dependence of the single-channel ILD thresholds, the frequency dependence for across-frequency binaural interference for ILDs currently has few compelling explanations (Rosen and Goupell, 2022).

The summary of the results for the older listeners is best understood in comparison to the younger listeners. Interferers at 0.5 and 1 kHz consistently had larger amounts of interference for the older listeners compared to the younger listeners (Figure 4, bottom two rows, below the solid line, highlighted by the number of boxes with red shading). Interferers at 2, 4, and 8 kHz had relatively smaller amounts of interference for the older listeners, which had smaller or comparable interference values compared to younger listeners (Figure 4, top three rows, above the solid line, highlighted by the number of boxes with blue shading). The boundary between 1 and 2 kHz is important to consider because the zero ITD of the stimuli may become particularly salient and important for stimuli <1.5 kHz. The binaural interference indices were the largest in the area that included the 0.5-, 1-, and 2-kHz interferer frequencies and the 2- and 4-kHz target frequencies (see Figure 4B, cluster of boxes with red diagonal lines). Since the binaural interference indices for the 4- and 8-kHz interferer frequencies were reduced for the older listeners compared to the younger listeners, this was consistent with the hypothesis about the relatively increasing importance of lower frequencies for older listeners, from either temporal processing deficits or hearing thresholds. Considering naturally occurring stimuli like speech, increasing the relative importance of lower-frequency ILDs could have some similarities to conditions in the present study; naturally occurring ILDs will trend toward zero in the low-frequency limit. This may be evidence for frequency-dependent plasticity to accommodate the aging auditory system. There were also significant interactions between age, target hearing thresholds, and interferer hearing thresholds, suggesting that the hearing thresholds at both the target and interferer frequencies contribute to the amount of interference.

Smith-Olinde et al. (1998) measured across-frequency binaural interference for three YNH (age range = 23–36 yrs) and six hearing-impaired (age range = 32–64 yrs) listeners using 1/3rd-octave narrowband noises centered at 0.5 and 4 kHz. Comparing the YNH listeners for the 0.5-kHz target and 4-kHz interferer, there was a 0.8-dB binaural interference index in that study and 0.7-dB binaural interference index in the current study (Figure 4), which is good correspondence. Comparing the YNH listeners for the 4-kHz target and 0.5-kHz interferer, there was a 0-dB binaural interference index in that study and a 0.7-dB binaural interference index in the current study. Comparing the hearing-impaired and ONH listeners for the 0.5-kHz target and 4-kHz interferer, there was a 0.2-dB binaural interference index in that study and 1.2-dB binaural interference index in the current study (Figure 4). Comparing the hearing-impaired and ONH listeners for the 4-kHz target and 0.5-kHz interferer, there was a 2.3-dB binaural interference index in that study and a 4.5-dB binaural interference index in the current study. Therefore, the current study often demonstrated more across-frequency binaural interference than in Smith-Olinde et al. (1998). The differences across studies may have been caused by differences in the stimuli [narrowband noises vs. tones (current study)], differences in level roving range [10 vs. 20 dB (current study)], and that study had some younger listeners with more hearing loss whereas the current study recruited older listeners with minimal hearing loss.

Few studies have considered the frequency dependence of sound localization or lateralization for both ITD and ILDs, as well as any age- or hearing-loss-related changes to such frequency dependence (Eddins and Hall, 2010; Gallun et al., 2021). It is clear that YNH listeners heavily weight low-frequency ITDs for broadband sound localization (Wightman and Kistler, 1992; Macpherson and Middlebrooks, 2002), particularly around 600–700 Hz (Bilsen and Raatgever, 1973; Stern et al., 1988). There are contributions from both ITDs and ILDs to intracranial lateralization when investigating how ITDs and ILDs derived from natural stimuli affect lateralization (Goupell and Stakhovskaya, 2018b). The contributions of ILDs are relatively small at lower frequencies compared to higher frequencies, which is partially a result of ILDs being physically smaller at low frequencies and that ILDs applied to lower frequencies produce less lateralization than ILDs applied to higher frequencies (Bernstein and Trahiotis, 2011; Goupell and Stakhovskaya, 2018b). For the stimuli in the present study, the zero ITDs could contribute to the changes in lateralization across frequency. Nevertheless, ITD and ILD reweighting appears to be possible with explicit training. For example, Klingel et al. (2021) found that YNH listeners reweighted ITD or ILD cues using 3-kHz noise bursts and seven days of audio-visual training. Keating et al. (2014) showed ferrets improved their 1-kHz ILD discrimination thresholds with explicit training, but not their 2-kHz thresholds, and was discussed in terms of reweighting of ITD and ILD cues. Humans can also accommodate changes to their spatial maps in response to changes in ITDs and ILDs induced by ear plugs (Kumpik et al., 2010). It is unclear what physiological center combines ITD and ILD information, and it is also unclear where across-frequency processing occurs, but it is possible that these centers are quite high, even cortical, if they readily experience plasticity. Sollini et al. (2017) found across-frequency processing of ILDs in the auditory cortex that was absent in the inferior colliculus.

Finally, one noteworthy condition in Figure 4B for the older listeners was the 8-kHz target, 4-kHz interferer condition. This condition had a binaural interference index of −0.8 dB (SD = 2.3 dB), which is a condition that could be demonstrating facilitation (i.e., negative interference). This value was not statistically lower than 0 dB (one-sample two-tailed *t*-test, *p* = 0.245), and therefore was is not distinguishable from measurement noise. Hence, no measurement to date has provided convincing evidence of across-frequency ILD-based facilitation (Bernstein and Trahiotis, 1995).

### Limitations and Future Directions

While the present study expands the understanding of the age-dependent frequency-dependent changes of across-frequency ILD processing, there are some clear future directions. First, as is done in many aging studies, older listeners with minimal hearing loss up to 2–4 kHz were recruited for the current study. Despite this, significant effects of hearing thresholds occurred in this current study, consistent with other reports showing even small changes in hearing thresholds affect binaural sensitivity (e.g., Bernstein and Trahiotis, 2016, 2018). An alternative approach would be to not limit the listener recruitment based on hearing thresholds or age (e.g., recruit younger listeners with hearing loss). Larger amounts of hearing loss and variability across frequency would help clarify the roles of hearing thresholds (i.e., assumed to be peripheral changes) and age (i.e., assumed to be central changes).

Given the effect of hearing thresholds, another future direction would be to systematically and parametrically investigate stimulus level effects. Smith-Olinde et al. (2004) measured across-frequency binaural interference for four YNH (age range = 27–39 yrs) and seven hearing-impaired listeners (age range = 40–66 yrs) at either equal sound pressure level or equal sensation level, compensating for the stimulus presentation level confound for the hearing-impaired listeners. Others have noted the importance of stimulus level in binaural perception tasks when testing hearing-impaired listeners (Häusler et al., 1983; Gallun et al., 2021). This is a clearly important variable for investigation, because across-frequency binaural interference studies have not systematically explored how the relative level across frequencies affects interference, despite how this should occur naturally in stimuli like speech and in listeners who have different configurations of hearing loss.

The frequency-specific average interaural hearing thresholds were significant predictive factors; the frequency-specific interaural difference in hearing thresholds (i.e., asymmetry) could be similarly considered. It was not possible to use this approach in the current study because there were mostly interaurally symmetric listeners. It is common in binaural-hearing studies to recruit interaurally symmetric listeners with ≤10 dB interaural hearing threshold difference. Interaural threshold asymmetry might induce plasticity that could be age dependent. Listeners with asymmetric hearing appear to perceive centered images with equal sound pressure levels presented to the two ears instead of equal sensation levels (Simon and Aleksandrovsky, 1997) and discrimination thresholds are often best at equal sound pressure levels (Hawkins and Wightman, 1980; Smoski and Trahiotis, 1986; Koehnke et al., 1995).

An across-frequency binaural interference paradigm was used in a highly controlled laboratory test, where some frequency bands have zero ITD and/or ILD. Natural sound sources away from the midline have combinations of non-zero ITDs and ILDs across frequencies. Future studies like Goupell and Stakhovskaya (2018b) that investigate age- and hearing-loss-related changes to lateralization or localization using interaural differences derived from natural stimuli would be another important future direction.

The current data could be interpreted as evidence for frequency-dependent plasticity as a listener ages. Another approach of assessing age-related plasticity would be to measure across-frequency binaural interference longitudinally so that the age-related and frequency-dependent differences in binaural interference are confirmed within the same listeners. There is a possibility that these patterns are partially a result of idiosyncratic listener tendencies, a hallmark of across-frequency binaural interference studies (Best et al., 2007, 2021), from a small set of listeners. Another alternative interpretation of the data is that older listeners have difficulty inhibiting distracting stimuli (Hasher et al., 1991). The physiological center where across-frequency binaural interference occurs is unknown, but given that release from interference that occurs when using grouping and streaming paradigms (Best et al., 2007), this suggests a higher (perhaps cortical) level, which would be amenable to a non-auditory cognitive explanation. An argument against a non-auditory age-related cognitive change is that there would be little reason to include frequency-dependent changes with age.

## Conclusions

The frequency dependence of across-frequency binaural interference was compared across younger and older listeners, all listeners having relatively good hearing thresholds for their age, but the older listeners had larger average audiometric thresholds compared to the younger listeners. ILD thresholds were worse for older compared to younger listeners and the amount of interference at specific target-interferer frequency combinations also increased. Frequency-specific hearing thresholds also contributed to the increased ILD thresholds and amount of interference. This provides some evidence for plasticity in the frequency weighting of binaural information.

## Data Availability Statement

The datasets presented in this study can be found in online repositories. The names of the repository/repositories and accession number(s) can be found at: https://doi.org/10.17605/OSF.IO/AF8M3.

## Ethics Statement

The studies involving human participants were reviewed and approved by the Institutional Review Board in the Division of Research. The patients/participants provided their written informed consent to participate in this study.

## Author Contributions

MG obtained funding for the study, designed the study, programmed the experiment, oversaw data collection, analyzed the data, and wrote the manuscript. All authors contributed to the article and approved the submitted version.

## Funding

Research reported in this publication was supported by the National Institute on Deafness and Other Communication Disorders of the National Institutes of Health under Award Number R01DC014948.

## Author Disclaimer

The content is solely the responsibility of the author and does not necessarily represent the official views of the National Institutes of Health.

## Conflict of Interest

The author declares that the research was conducted in the absence of any commercial or financial relationships that could be construed as a potential conflict of interest.

## Publisher's Note

All claims expressed in this article are solely those of the authors and do not necessarily represent those of their affiliated organizations, or those of the publisher, the editors and the reviewers. Any product that may be evaluated in this article, or claim that may be made by its manufacturer, is not guaranteed or endorsed by the publisher.

## References

[B1] Anderson S. Bieber R. Schloss A. (2021). Peripheral deficits and phase-locking declines in aging adults. Hear. Res. 403, 108188. 10.1016/j.heares.2021.108188 33581668 PMC7980782

[B2] Anderson S. Ellis R. Mehta J. Goupell M. J. (2018). Age-related differences in binaural masking level differences: behavioral and electrophysiological evidence. J. Neurophysiol. 120, 2939–2952. 10.1152/jn.00255.2018 30230989 PMC6337034

[B3] Anderson S. R. Easter K. Goupell M. J. (2019). Effects of rate and age in processing interaural time and level differences in normal-hearing and bilateral cochlear-implant listeners. J. Acoust. Soc. Am. 146, 3232–3254. 10.1121/1.5130384 31795662 PMC6948219

[B4] Ashida G. Tollin D. J. Kretzberg J. (2021). Robustness of neuronal tuning to binaural sound localization cues against age-related loss of inhibitory synaptic inputs. PLoS Comput. Biol. 17, e1009130. 10.1371/journal.pcbi.1009130 34242210 PMC8270189

[B5] Babkoff H. Muchnik C. Ben-David N. Furst M. Even-Zohar S. Hildesheimer M. (2002). Mapping lateralization of click trains in younger and older populations. Hear. Res. 165, 117–127. 10.1016/S0378-5955(02)00292-7 12031521

[B6] Bates D. Maechler M. Bolker B. Walker S. (2015). Fitting linear mixed-effects models using lme4. J. Stat. Softw. 67, 1–48. 10.18637/jss.v067.i01

[B7] Bernstein L. R. Trahiotis C. (1995). Binaural interference effects measured with masking-level difference and with ITD- and IID-discrimination paradigms. J. Acoust. Soc. Am. 98, 155–163. 10.1121/1.414467 7608395

[B8] Bernstein L. R. Trahiotis C. (2011). Lateralization produced by interaural intensitive disparities appears to be larger for high- vs low-frequency stimuli. J. Acoust. Soc. Am. 1, EL15–EL20. 10.1121/1.3528756 21302976 PMC3037973

[B9] Bernstein L. R. Trahiotis C. (2016). Behavioral manifestations of audiometrically-defined “slight” or “hidden” hearing loss revealed by measures of binaural detection. J. Acoust. Soc. Am. 140, 3540–3548. 10.1121/1.4966113 27908080

[B10] Bernstein L. R. Trahiotis C. (2018). Effects of interaural delay, center frequency, and no more than “slight” hearing loss on precision of binaural processing: empirical data and quantitative modeling. J. Acoust. Soc. Am. 144, 292–307. 10.1121/1.5046515 30075692

[B11] Bernstein L. R. Trahiotis C. (2021). A crew of listeners with no more than “slight” hearing loss who exhibit binaural deficits also exhibit reduced amounts of binaural interference. J. Acoust. Soc. Am. 150, 2977–2984. 10.1121/10.0006733 34717472

[B12] Best V. Gallun F. J. Carlile S. Shinn-Cunningham B. G. (2007). Binaural interference and auditory grouping. J. Acoust. Soc. Am. 121, 1070–1076. 10.1121/1.2407738 17348529

[B13] Best V. Goupell M. J. Colburn H. S. (2021). “Binaural Hearing and Across-Channel Processing,” in Binaural Hearing, eds R. Y. Litovsky, M. J. Goupell, R. R. Fay, and A. N. Popper (Cham: Springer International Publishing), 181–207.

[B14] Best V. Swaminathan J. (2019). Revisiting the detection of interaural time differences in listeners with hearing loss. J. Acoust. Soc. Am. 145, EL508–EL513. 10.1121/1.5111065 31255153 PMC6561774

[B15] Bibee J. M. Stecker G. C. (2016). Spectrotemporal weighting of binaural cues: effects of a diotic interferer on discrimination of dynamic interaural differences. J. Acoust. Soc. Am. 140, 2584–2592. 10.1121/1.4964708 27794286 PMC5849029

[B16] Bilsen F. A. Raatgever J. (1973). Spectral dominance in lateralization. Acustica 28, 131–132.

[B17] Brown A. D. Tollin D. J. (2016). Slow temporal integration enables robust neural coding and perception of a cue to sound source location. J. Neurosci. 36, 9908–9921. 10.1523/JNEUROSCI.1421-16.2016 27656028 PMC5030352

[B18] Brown A. D. Tollin D. J. (2021). Effects of interaural decoherence on sensitivity to interaural level differences across frequency. J. Acoust. Soc. Am. 149, 4630–4648. 10.1121/10.0005123 34241434 PMC8249038

[B19] Brughera A. Dunai L. Hartmann W. M. (2013). Human interaural time difference thresholds for sine tones: the high-frequency limit. J. Acoust. Soc. Am. 133, 2839–2855. 10.1121/1.4795778 23654390 PMC3663869

[B20] Casey M. A. (1990). The effects of aging on neuron number in the rat superior olivary complex. Neurobiol. Aging 11, 391–394. 10.1016/0197-4580(90)90004-J 2381498

[B21] Cecato J. F. Martinelli J. E. Izbicki R. Yassuda M. S. Aprahamian I. (2016). A subtest analysis of the Montreal cognitive assessment (MoCA): which subtests can best discriminate between healthy controls, mild cognitive impairment and Alzheimer's disease? Int. Psychogeriatr. 28, 825–832. 10.1017/S1041610215001982 26620850

[B22] Devries L. Anderson S. Goupell M. J. Smith E. Gordon-Salant S. (2022). Effects of aging and hearing loss on perceptual and electrophysiological pulse rate discrimination. J. Acoust. Soc. Am. 151, 1639–1650. 10.1121/10.0009399 35364956 PMC8916844

[B23] Dobreva M. S. O'neill W. E. Paige G. D. (2011). Influence of aging on human sound localization. J. Neurophysiol. 105, 2471–2486. 10.1152/jn.00951.2010 21368004 PMC3094163

[B24] Dupuis K. Pichora-Fuller M. K. Chasteen A. L. Marchuk V. Singh G. Smith S. L. (2015). Effects of hearing and vision impairments on the Montreal Cognitive Assessment. Neuropsychol. Dev. Cogn. B Aging Neuropsychol. Cogn. 22, 413–437. 10.1080/13825585.2014.968084 25325767

[B25] Durlach N. I. Thompson C. L. Colburn H. S. (1981). Binaural interaction of impaired listeners. A review of past research. Audiology 20, 181–211. 10.3109/00206098109072694 7011289

[B26] Eddins A. C. Eddins D. A. (2018). Cortical correlates of binaural temporal processing deficits in older adults. Ear Hear. 39, 594–604. 10.1097/AUD.0000000000000518 29135686 PMC5920708

[B27] Eddins A. C. Ozmeral E. J. Eddins D. A. (2018). How aging impacts the encoding of binaural cues and the perception of auditory space. Hear. Res. 369, 79–89. 10.1016/j.heares.2018.05.001 29759684 PMC6196106

[B28] Eddins D. A. Hall J. W. (2010). “Binaural processing and auditory asymmetries,” in The Aging Auditory System, eds S. Gordon-Salant, D. R. Frisina, A. N. Popper, and R. R. Fay (New York, NY: Springer), 135–165.

[B29] Feddersen W. E. Sandel T. T. Teas D. C. Jeffress L. A. (1957). Localization of high-frequency tones. J. Acoust. Soc. Am. 29, 988–991. 10.1121/1.1909356

[B30] Finlayson P. G. (1995). Decreased inhibition to lateral superior olive neurons in young and aged Sprague-Dawley rats. Hear. Res. 87, 84–95. 10.1016/0378-5955(95)00081-E 8567446

[B31] Finlayson P. G. Caspary D. M. (1993). Response properties in young and old Fischer-344 rat lateral superior olive neurons: a quantitative approach. Neurobiol. Aging 14, 127–139. 10.1016/0197-4580(93)90088-S 8487915

[B32] Franken T. P. Joris P. X. Smith P. H. (2018). Principal cells of the brainstem's interaural sound level detector are temporal differentiators rather than integrators. Elife 7, e33854. 10.7554/eLife.33854.027 29901438 PMC6063729

[B33] Füllgrabe C. Moore B. C. J. (2018). The association between the processing of binaural temporal-fine-structure information and audiometric threshold and age: a meta-analysis. Trends Hear. 22, 2331216518797259. 10.1177/2331216518797259 30261828 PMC6166311

[B34] Füllgrabe C. Sek A. P. Moore B. C. J. (2018). Senescent changes in sensitivity to binaural temporal fine structure. Trends Hear. 22, 2331216518788224. 10.1177/2331216518788224 30027803 PMC6055238

[B35] Gabriel K. J. Koehnke J. Colburn H. S. (1992). Frequency dependence of binaural performance in listeners with impaired binaural hearing. J. Acoust. Soc. Am. 91, 336–347. 10.1121/1.402776 1737882

[B36] Gallun F. J. (2021). Impaired binaural hearing in adults: a selected review of the literature. Front. Neurosci. 15, 610957. 10.3389/fnins.2021.610957 33815037 PMC8017161

[B37] Gallun F. J. Best V. (2020). “Age-related changes in segregation of sound sources,” in Aging and Hearing: Causes and Consequences, eds K. S. Helfer, E. L. Bartlett, A. N. Popper, and R. R. Fay (Cham: Springer International Publishing), 143–171.

[B38] Gallun F. J. Srinivasan N. K. Diedesch A. C. (2021). “Clinical ramifications of the effects of hearing impairment and aging on spatial and binaural hearing,” in Binaural Hearing, eds R. Y. Litovsky, M. J. Goupell, R. R. Fay, and A. N. Popper (Cham: Springer International Publishing), 317–347.

[B39] Goldberg J. M. Brown P. B. (1969). Response of binaural neurons of dog superior olivary complex to dichotic tonal stimuli: some physiological mechanisms of sound localization. J. Neurophysiol. 32, 613–636. 10.1152/jn.1969.32.4.613 5810617

[B40] Gordon-Salant S. Frisina R. D. Popper A. N. Fay R. R. (2010). The Aging Auditory System. New York, NY: Springer.

[B41] Goupell M. J. Stakhovskaya O. A. (2018a). Across-channel interaural-level-difference processing demonstrates frequency dependence. J. Acoust. Soc. Am. 143, 645–658. 10.1121/1.5021552 29495743 PMC5798994

[B42] Goupell M. J. Stakhovskaya O. A. (2018b). Across-frequency processing of interaural time and level differences in perceived lateralization. Acta Acust. Acust. 104, 758–761. 10.3813/AAA.919217 31093031 PMC6513309

[B43] Grantham D. W. (1984). Interaural intensity discrimination: insensitivity at 1000 Hz. J. Acoust. Soc. Am. 75, 1191–1194. 10.1121/1.390769 6725768

[B44] Grose J. H. Mamo S. K. (2012). Frequency modulation detection as a measure of temporal processing: age-related monaural and binaural effects. Hear. Res. 294, 49–54. 10.1016/j.heares.2012.09.007 23041187 PMC3505233

[B45] Hartmann W. M. (2021). “Localization and lateralization of sound,” in Binaural Hearing, eds R. Y. Litovsky, M. J. Goupell, R. R. Fay, and A. N. Popper (Cham: Springer International Publishing), 9–45.

[B46] Hartmann W. M. Constan Z. A. (2002). Interaural level differences and the level-meter model. J. Acoust. Soc. Am. 112, 1037–1045. 10.1121/1.1500759 12243152

[B47] Hasher L. Stoltzfus E. R. Zacks R. T. Rypma B. (1991). Age and inhibition. J. Exp. Psychol. Learn. Mem. Cogn. 17, 163–169. 10.1037/0278-7393.17.1.163 1826730

[B48] Häusler R. Colburn S. Marr E. (1983). Sound localization in subjects with impaired hearing. spatial-discrimination and interaural-discrimination tests. Acta Otolaryngol. Suppl. 96, 1–62. 10.3109/00016488309105590 6316714

[B49] Hawkins D. B. Wightman F. L. (1980). Interaural time discrimination ability of listeners with sensorineural hearing loss. Audiology 19, 495–507. 10.3109/00206098009070081 7425954

[B50] Helfer K. S. Bartlett E. Popper A. N. Fay R. R. (2020). Aging and Hearing: Causes and Consequences. Cham: Springer Nature Switzerland.

[B51] Heller L. M. Richards V. M. (2010). Binaural interference in lateralization thresholds for interaural time and level differences. J. Acoust. Soc. Am. 128, 310–319. 10.1121/1.3436524 20649226 PMC2921431

[B52] Herman G. E. Warren L. R. Wagener J. W. (1977). Auditory lateralization: age differences in sensitivity to dichotic time and amplitude cues. J. Gerontol. 32, 187–191. 10.1093/geronj/32.2.187

[B53] Ihlefeld A. Carlyon R. P. Kan A. Churchill T. H. Litovsky R. Y. (2015). Limitations on monaural and binaural temporal processing in bilateral cochlear implant listeners. J. Assoc. Res. Otolaryngol. 16, 641–652. 10.1007/s10162-015-0527-7 26105749 PMC4569611

[B54] Jones H. G. Brown A. D. Koka K. Thornton J. L. Tollin D. J. (2015). Sound frequency-invariant neural coding of a frequency-dependent cue to sound source location. J. Neurophysiol. 114, 531–539. 10.1152/jn.00062.2015 25972580 PMC4509402

[B55] Keating P. Nodal F. R. King A. J. (2014). Behavioural sensitivity to binaural spatial cues in ferrets: evidence for plasticity in the duplex theory of sound localization. Eur. J. Neurosci. 39, 197–206. 10.1111/ejn.12402 24256073 PMC4063341

[B56] Klingel M. Kopčo N. Laback B. (2021). Reweighting of binaural localization cues induced by lateralization training. J. Assoc. Res. Otolaryngol. 22, 551–566. 10.1007/s10162-021-00800-8 33959826 PMC8476684

[B57] Koehnke J. Culotta C. P. Hawley M. L. Colburn H. S. (1995). Effects of reference interaural time and intensity differences on binaural performance in listeners with normal and impaired hearing. Ear Hear. 16, 331–353. 10.1097/00003446-199508000-00001 8549890

[B58] Kumpik D. P. Kacelnik O. King A. J. (2010). Adaptive reweighting of auditory localization cues in response to chronic unilateral earplugging in humans. J. Neurosci. 30, 4883–4894. 10.1523/JNEUROSCI.5488-09.2010 20371808 PMC4225134

[B59] Laback B. Dietz M. Joris P. (2017). Temporal effects in interaural and sequential level difference perception. J. Acoust. Soc. Am. 142, 3267–3283. 10.1121/1.5009563 29195428

[B60] Laumen G. Tollin D. J. Beutelmann R. Klump G. M. (2016). Aging effects on the binaural interaction component of the auditory brainstem response in the Mongolian gerbil: effects of interaural time and level differences. Hear. Res. 337, 46–58. 10.1016/j.heares.2016.04.009 27173973 PMC4922418

[B61] Levitt H. (1971). Transformed up-down methods in psychoacoustics. J. Acoust. Soc. Am. 49, 467–477. 10.1121/1.1912375 5541744

[B62] Macaulay E. J. Hartmann W. M. Rakerd B. (2010). The acoustical bright spot and mislocalization of tones by human listeners. J. Acoust. Soc. Am. 127, 1440–1449. 10.1121/1.3294654 20329844 PMC2856510

[B63] Macpherson E. A. Middlebrooks J. C. (2002). Listener weighting of cues for lateral angle: the duplex theory of sound localization revisited. J. Acoust. Soc. Am. 111, 2219–2236. 10.1121/1.1471898 12051442

[B64] Mcfadden D. Pasanen E. G. (1976). Lateralization of high frequencies based on interaural time differences. J. Acoust. Soc. Am. 59, 634–639. 10.1121/1.380913 1254790

[B65] Mills A. W. (1958). On the minimum audible angle. J. Acoust. Soc. Am. 30, 237–246. 10.1121/1.1909553

[B66] Mills A. W. (1960). Lateralization of high-frequency tones. J. Acoust. Soc. Am. 32, 132–134. 10.1121/1.1907864

[B67] Nasreddine Z. S. Phillips N. A. Bédirian V. Charbonneau S. Whitehead V. Collin I.. (2005). The Montreal Cognitive Assessment, MoCA: a brief screening tool for mild cognitive impairment. J. Am. Geriatr. Soc. 53, 695–699. 10.1111/j.1532-5415.2005.53221.x 15817019

[B68] Otte R. J. Agterberg M. J. Van Wanrooij M. M. Snik A. F. Van Opstal A. J. (2013). Age-related hearing loss and ear morphology affect vertical but not horizontal sound-localization performance. J. Assoc. Res. Otolaryngol. 14, 261–273. 10.1007/s10162-012-0367-7 23319012 PMC3660912

[B69] Ozmeral E. J. Eddins D. A. Eddins A. C. (2016). Reduced temporal processing in older, normal-hearing listeners evident from electrophysiological responses to shifts in interaural time difference. J. Neurophysiol. 116, 2720–2729. 10.1152/jn.00560.2016 27683889 PMC5133308

[B70] R Development Core Team (2021). R: A Language and Environment for Statistical Computing. Vienna. Available online at: https://www.R-project.org/ (accessed February 13, 2022).

[B71] Rosen B. Goupell M. J. (2022). The effect of target and interferer frequency on across-frequency binaural interference of interaural-level-difference sensitivity. J. Acoust. Soc. Am. 151, 924–938. 10.1121/10.0009398 35232088 PMC8837388

[B72] Ross B. Fujioka T. Tremblay K. L. Picton T. W. (2007). Aging in binaural hearing begins in mid-life: evidence from cortical auditory-evoked responses to changes in interaural phase. J. Neurosci. 27, 11172–11178. 10.1523/JNEUROSCI.1813-07.2007 17942712 PMC6673023

[B73] Simon H. J. Aleksandrovsky I. (1997). Perceived lateral position of narrow-band noise in hearing-impaired and normal-hearing listeners under conditions of equal sensation level and sound-pressure level. J. Acoust. Soc. Am. 102, 1821–1826. 10.1121/1.420089 9301059

[B74] Smith-Olinde L. Besing J. Koehnke J. (2004). Interference and enhancement effects on interaural time discrimination and level discrimination in listeners with normal hearing and those with hearing loss. Am. J. Audiol. 13, 80–95. 10.1044/1059-0889(2004/011) 15248807

[B75] Smith-Olinde L. Koehnke J. Besing J. (1998). Effects of sensorineural hearing loss on interaural discrimination and virtual localization. J. Acoust. Soc. Am. 103, 2084–2099. 10.1121/1.421355 9566330

[B76] Smoski W. J. Trahiotis C. (1986). Discrimination of interaural temporal disparities by normal-hearing listeners and listeners with high-frequency sensorineural hearing loss. J. Acoust. Soc. Am. 79, 1541–1547. 10.1121/1.393680 3711453

[B77] Sollini J. Mill R. Sumner C. J. (2017). Spatial processing is frequency specific in auditory cortex but not in the midbrain. J. Neurosci. 37, 6588–6599. 10.1523/JNEUROSCI.3034-16.2017 28559383 PMC5511886

[B78] Spencer N. J. Hawley M. L. Colburn H. S. (2016). Relating interaural difference sensitivities for several parameters measured in normal-hearing and hearing-impaired listeners. J. Acoust. Soc. Am. 140, 1783–1799. 10.1121/1.4962444 27914394 PMC5035301

[B79] Srinivasan N. K. Jakien K. M. Gallun F. J. (2016). Release from masking for small spatial separations: effects of age and hearing loss. J. Acoust. Soc. Am. 140, EL73–EL78. 10.1121/1.4954386 27475216 PMC5392088

[B80] Stern R. M. Zeiberg A. S. Trahiotis C. (1988). Lateralization of complex binaural stimuli: a weighted-image model. J. Acoust. Soc. Am. 84, 156–165. 10.1121/1.396982 3411043

[B81] Stevens S. S. Newman E. B. (1936). The localization of actual sources of sound. Am. J. Psychol. 48, 297–306. 10.2307/1415748

[B82] Strouse A. Ashmead D. H. Ohde R. N. Grantham D. W. (1998). Temporal processing in the aging auditory system. J. Acoust. Soc. Am. 104, 2385–2399. 10.1121/1.423748 10491702

[B83] Strutt J. W. (1907). On our perception of sound direction. Phil. Mag. 13, 214–232. 10.1080/14786440709463595

[B84] Voeten C. C. (2020). Buildmer: Stepwise Elimination and Term Reordering for Mixed-Effects Regression, 1.7.1 Edn. Available online at: https://rdrr.io/cran/buildmer/

[B85] Wightman F. L. Kistler D. J. (1992). The dominant role of low-frequency interaural time differences in sound localization. J. Acoust. Soc. Am. 91, 1648–1661. 10.1121/1.402445 1564201

[B86] Working Group on Speech Understanding and Aging (1988). Speech understanding and aging. J. Acoust. Soc. Am. 83, 859–895. 10.1121/1.395965 3281988

[B87] Yin T. C. T. Chan J. C. (1990). Interaural time sensitivity in medial superior olive of cat. J. Neurophysiol. 64, 465–488. 10.1152/jn.1990.64.2.465 2213127

[B88] Yost W. A. Dye R. H. Jr. (1988). Discrimination of interaural differences of level as a function of frequency. J. Acoust. Soc. Am. 83, 1846–1851. 10.1121/1.396520 3403800

